# Differential Lipidomic Characteristics of Children Born to Women with Polycystic Ovary Syndrome

**DOI:** 10.3389/fendo.2021.698734

**Published:** 2021-08-09

**Authors:** Zhirong Zhang, Yue Liu, Jiali Lv, Di Zhang, Kuona Hu, Jingyu Li, Jinlong Ma, Linlin Cui, Han Zhao

**Affiliations:** ^1^Center for Reproductive Medicine, Cheeloo College of Medicine, Shandong University, Jinan, China; ^2^Key laboratory of Reproductive Endocrinology of Ministry of Education, Shandong University, Jinan, China; ^3^Shandong Key Laboratory of Reproductive Medicine, Shandong University, Jinan, China; ^4^Shandong Provincial Clinical Research Center for Reproductive Health, Shandong University, Jinan, China; ^5^National Research Center for Assisted Reproductive Technology and Reproductive Genetics, Shandong University, Jinan, China; ^6^Department of Biostatistics, School of Public Health, Cheeloo College of Medicine, Shandong University, Jinan, China

**Keywords:** lipid metabolism, lipidomics, polycystic ovary syndrome, offspring, cardiometabolic health

## Abstract

**Objective:**

To describe the lipidomic characteristics of offspring born to polycystic ovary syndrome (PCOS) women (PCOS-off) and assess the associations between differential lipids and clinical phenotypes.

**Methods:**

Ultra performance liquid chromatography and mass spectrometry were performed on plasma samples from 70 PCOS-off and 71 healthy controls. The associations of differential metabolites with clinical phenotypes were examined by multiple linear regression.

**Results:**

Forty-four metabolites were significantly altered in PCOS-off, including 8 increased and 36 decreased. After stratification according to sex, 44 metabolites (13 increased and 31 decreased) were expressed differently in girls born to PCOS women (PCOS-g), most of which were glycerolipids. Furthermore, 46 metabolites (9 increased and 35 decreased) were expressed differently in boys born to PCOS women (PCOS-b), most of which were glycerophospholipids. Significant associations of metabolites with weight Z-score and high density lipoprotein cholesterol were found in PCOS-off. Triglycerides, low density lipoprotein cholesterol, and thyroid-stimulating hormone were separately correlated with some lipids in PCOS-g and PCOS-b.

**Conclusions:**

PCOS-off showed specific lipid profile alterations. The abnormal level of glycerophospholipids and sphingomyelin indicated the risk of glucose metabolism and cardiovascular diseases in PCOS-off. Some lipids, such as phosphatidylcholines, lysophosphatidylcholine and sphingomyelin, may be the potential markers. The results broadened our understanding of PCOS-offs’ cardiometabolic status and emphasized more specific and detailed monitoring and management in this population.

## Introduction

As the most prevalent endocrine disorder in women of fertile age, polycystic ovary syndrome (PCOS) is known to be associated with several adverse metabolic outcomes, including obesity, dyslipidemia, insulin resistance, and type 2 diabetes (T2D) (1–3). In addition, women with PCOS are at increased risk of pregnancy complications, such as pregnancy-induced hypertension (PIH), gestational diabetes mellitus (GDM), pre-eclampsia, and premature delivery (4, 5). Aberrant metabolic pathophysiological changes and increased pregnancy complications predispose PCOS women towards suboptimal intrauterine environments (2, 6–8). According to the Barker hypothesis, this may produce a detrimental impact on the cardiometabolic health of offspring born to PCOS women (PCOS-off) (9–12). Previous studies report multiple metabolic phenotypic changes in PCOS-off, including dyslipidemia (12, 13), but others have contradictory findings. Some studies find that triglyceride (TG), cholesterol (CHOL), high density lipoprotein cholesterol (HDL), and adiponectin levels in the cord blood of newborns of PCOS women are similar to those of controls (14, 15).

Metabolomics allows for large-scale qualitative and semi-quantitative analysis of numerous nutrients, small molecules, and metabolic intermediates and has proven to be a powerful tool for identifying alterations in metabolic pathways and novel biomarkers (16). As a branch of metabolomics, lipidomics offers opportunities to investigate a large range of lipids in biological tissues and fluids. Lipids are accepted to be directly involved in the pathogenesis of cardiovascular disorders, and are thus proposed as independent predictive markers (17, 18). Therefore, having a global view of lipid metabolism in PCOS-off will improve our understanding of the cardiometabolic health of this susceptible population and identify new biomarkers for early detection of metabolic aberrations.

The aim of this study was to assess lipid profiles in PCOS-off and determine the associations of plasma lipids in PCOS-off with their clinical phenotypes. The results could broaden our understanding of PCOS-offs’ cardiometabolic status and provide support for long-term monitoring and management.

## Materials and Methods

### Study Populations

Children of PCOS women and control women were recruited in the clinics of Center for Reproductive Medicine, Shandong University based on a cohort study. PCOS diagnosis was defined according to the Rotterdam criteria. The inclusion criteria were women with two out of the following three characteristics, oligo-ovulation, clinical and/or biochemical hyperandrogenism and ovarian polycystic morphology in ultrasound examination. Patients with other causes of hyperandrogenism or oligomenorrhea, such as thyroid disease, hyperprolactinemia and Cushing’s syndrome, were excluded. The detailed diagnostic procedure was described in our previous study (12). A total of 141 children were enrolled with 70 PCOS-off and 71 controls. The control offspring were recruited according to their mothers’ age and BMI which were matched with the case group. The age of participated children ranged from 2.5 to 7.0 years old. Growth and development parameters were available in all children, including birth weight, gestational age, height, weight, diastolic blood pressure (DBP), systolic blood pressure (SBP). Blood samples from all children were available and used to measure endocrine and metabolic parameters, including follicle-stimulating hormone (FSH), luteinizing hormone (LH), estrogen (E2), prolactin (PRL), testosterone (T), thyroid-stimulating hormone (TSH), anti-Mu ¨llerian hormone (AMH), dehydroepiandrosterone sulfate (DHEA-S), free triiodothyronine (FT3), free thyroxine (FT4), antibodies against thyroglobulin (A-TG), antibodies against thyroperoxidase (A-TPO), CHOL, fasting glucose (BG0), fasting insulin (INS0), TG, low density lipoprotein cholesterol (LDL), and HDL. The measurement methods and some calculation formulas, including BMI, the Z-score, homeostasis model for insulin resistance (HOMA-IR), and homeostasis model for β-cell function (HOMA-β), refer to our previous article (12).

### Sample Processing

The blood samples were obtained in the morning with EDTA anticoagulant tube, and then placed at room temperature for 2h. Thereafter, the blood samples were centrifuged at 2000 rpm, 4°C for 20 min, and the upper plasma was collected and stored at -80°C for lipidomics analysis.

### Lipidomics Analysis

#### Lipid Extraction

100μl of plasma samples were transferred to a tube, and then added 900 μl of water, 2 ml of methanol and 0.9 ml of dichloromethane followed by vortexing. After adding 1.0 ml of water and 0.9 ml of dichloromethane, the mixture was emulsified by vortexing and then centrifuged at 3000 rpm, 4°C for 5 min. The lower dichloromethane layer was injected to a clean glass tube using a glass syringe. 2 ml of dichloromethane was added to the supernatants and centrifuged at 3000 rpm, 4°C for 5 min. Then the dichloromethane layers collected by twice centrifugations were mixed and evaporated to dryness in a vacuum concentrator. The residue was redissolved in 500μl of methanol/dichloromethane and centrifuged at 3000 rpm, 4°C for 5 min. Finally, the supernatant was collected for LC-MS analysis and the same amount of supernatant from each sample was collected and mixed as QC sample.

#### Ultra Performance Liquid Chromatography Analysis

The Waters ACQUITY UPLC I-Class system was used for chromatographic analysis. By using a 20 min linear gradient, samples were transferred onto a C18 CSH column (100 mm × 2.1 mm, 1.7 μm; Waters) at 45°C at a flow rate of 0.4 mL/min and then chromatographic gradient elution procedure was performed. Mobile phase buffer A consisted of acetonitrile/water (1/4), 0.1% formic acid, and 10 mM ammonium format, and buffer B consisted of acetonitrile/isopropanol (1/9), 0.1% formic acid, and10 mM ammonium format.

#### Mass Spectrometry Analysis

The Xevo G2-S Q-TOF with an electrospray ionization (ESI) source (Waters, Manchester, UK) was used for mass spectrometry analysis. MS parameters were set as follows, ion source temperature, 100°C; desolvation temperature, 400°C; capillary voltage, 2.5 kV; cone voltage, 24 kV; cone gas flow, 50 L/h; and desolvation gas flow, 800 L/h (positive ion-mode); ion source temperature, 100°C; desolvation temperature, 500°C; capillary voltage, 2.5 kV; cone voltage, 25 kV; cone gas flow, 10 L/h; and desolvation gas flow, 600 L/h (negative ion-mode). Scan range of both modes was 100 to 1500m/z.

#### Data Processing and Metabolite Identification

The raw data were processed using the Progenesis QI (Waters) software and the peak alignment, peak picking, and quantitation were performed by the parameters of each compound, including m/z, retention time, and peak area. The relative quantitative and accurate qualitative results of metabolites were obtained by matching the data with database including NIST (https://chemdata.nist.gov), HMDB (http://www.hmdb.ca), lipidmaps (http://www.lipidmaps.org), and an in-house lipid database. The mass deviation was 5 ppm. Then the compounds with coefficient of variance < 30% in QC samples and detectable in at least 50% of samples were selected as the final data for subsequent analysis. The data were then logarithm transformed and standardized using MetaX software. Multivariate statistical analysis, including principal component analysis (PCA) and partial least-squares discrimination analysis (PLS-DA), were performed to reduce the data dimension and conduct regression analysis. Variable importance in the projection (VIP) value from PLS-DA model, fold change (FC), and p value of t-test were used to identify differential metabolites. The threshold value was set as VIP > 1.0, FC > 1.2 or FC < 0.833 and p value < 0.05. After that, cluster analysis and volcano plots were used to show the metabolic patterns and overall distribution of metabolites of interest.

### Statistical Analysis

All statistical analysis was performed using SPSS v26.0 software (SPSS Inc., Chicago, IL, USA). The tests of normality were performed by Kolmogorov Smirnov normality test, histogram, and Q-Q diagram. Continuous variables were presented as mean ± SD or median (interquartile range) according to the results of normality test. Categorical variables were presented as numbers (percentiles). Clinical characteristics were compared between study groups using t test, Mann–Whitney U test, or chi-square test.

In order to evaluate the associations between offspring phenotypes and differential metabolites, multiple linear regression analyses were performed, treating differential metabolites after natural logarithm transformation as predictors, and offspring phenotypes as outcomes. The missing values of metabolites were replaced by the minimum quantitative value of metabolites in all samples divided by twenty. Model 1 was an unadjusted model. Model 2 was adjusted for some anthropometric factors, including sex, age, and BMI. Model 3 was added some pregnancy and perinatal covariates (birth weight, mode of delivery, parity, and gestational age at delivery) based on model 2. Model 4 corrected all above variables plus other maternal and familial related factors, including maternal age, maternal BMI, education level, family monthly income (≤ ¥2999 = low, ¥3000-4999 = medium, ≥ ¥5000 = high), GDM, and PIH. Sex of offspring was not adjusted after gender stratification. Birth weight and children BMI were not adjusted in the linear regression analysis of body weight Z-score.

### Ethical Approval

This study was approved by the institutional ethics committee of Shandong University. The ethics approval number was (NO. 2014[17]). The parents of the children signed the informed consent. The study conformed to the principles of the Declaration of Helsinki.

## Results

### Clinical Characteristics

Baseline characteristics of the study population were summarized in **Table 1**. The study subjects consisted of 70 PCOS-off and 71 healthy controls with the percentages of girls in each group as 61.4% and 62.0% respectively. PCOS women presented with significantly higher LH, E2, and testosterone, as well as lower FSH levels. No statistical difference was observed in terms of maternal age, BMI, parity, cesarean section rate, and the incidence of PIH and GDM.

**Table 1:** Baseline characteristics of the study population.

Characteristics	PCOS-off (n=70)	Con-off (n=71)	*P* value
Maternal characteristics			
Age at delivery, yrs	28.8 ± 3.1	28.8 ± 3.2	0.97
BMI before pregnancy, kg/m^2^	24.3 ± 3.5	24.2 ± 3.2	0.90
University degree or higher, n (%)	21 (30.0%)	19 (26.8%)	0.67
T, ng/dl	40.5 (28.2-59.5)	29.3 (17.8-36.6)	<0.001^*^
FSH, IU/L	5.9 (5.1-6.7)	6.6 (5.5-7.6)	0.001^*^
LH, IU/L	7.8 (5.7-11.2)	4.8 (3.4-6.2)	<0.001^*^
E2, pg/ml	39.9 (30.9-49.9)	33.2 (24.7-41.8)	0.004^*^
Paternal characteristics			
Age at delivery, yrs	30.2 ± 4.2	29.6 ± 3.2	0.33
BMI before pregnancy, kg/m^2^	22.7 ± 1.8	22.9 ± 1.6	0.49
Parity			>0.999
1, n (%)	67 (95.7%)	67 (94.4%)	
≥2, n (%)	3 (4.3%)	4 (5.6%)	
Household income			0.25
Low, n (%)	44 (62.9%)	35 (49.3%)	
Medium, n (%)	20 (28.6%)	29 (40.8%)	
High, n (%)	6 (8.6%)	7 (9.9%)	
Cesarean, n (%)	51 (72.9%)	55 (77.5%)	0.53
PIH, n (%)	5 (7.1%)	2 (2.8%)	0.43
GDM, n (%)	2 (2.9%)	2 (2.8%)	>0.999
Female offspring, n (%)	43 (61.4%)	44 (62.0%)	0.95
Age of offspring, yrs	3.51 (3.20-5.16)	3.59 (3.19-5.05)	0.995

Data were shown as mean ± SD, median (interquartile range) or n (%).

Differences were assessed using Student t test or Mann-Whitney U tests for continuous variables and Chi-square tests for categorical variables.

^*^p<0.05 was considered statistically significant.

PCOS-off, offspring born to PCOS women; Con-off, offspring of the control group; BMI, body mass index; T, Testosterone; FSH, follicle-stimulating hormone; LH, luteinizing hormone; E2, estrogen; PIH, Pregnancy-induced hypertension; GDM, gestational diabetes mellitus.

**Table 2** showed the clinical characteristics of PCOS-off. PCOS-off tended to have lower weight Z-score. Other clinical characteristics were similar between the two groups. After stratification according to sex, boys born to PCOS women (PCOS-b) showed lower TSH level compared with their counterparts (**Table S1**). There was no difference in other clinical features in PCOS-b, nor were all characteristics in girls born to PCOS women (PCOS-g) (**Table S2**).

**Table 2:** Clinical characteristics of the general offspring born to women with or without PCOS.

Characteristics	PCOS-off (n=70)	Con-off (n=71)	*P* value
BW, g	3404.0 ± 570.8	3423.2 ± 446.4	0.82
GA, week	39.0 (38.0-40.0)	39.0 (38.0-40.0)	0.90
Height Z-score	0.3 ± 0.9	0.5 ± 0.8	0.27
Weight Z-score^a^	0.3 ± 1.0	0.7 ± 1.1	0.03^*^
BMI Z-score	0.2 (-0.5-0.6)	0.4 (-0.5-1.3)	0.10
DBP, mmHg^†^	58.0 (54.0-60.0)	58.0 (52.0-60.0)	0.51
SBP, mmHg^†^	90.0 (88.0-96.0)	90.0 (88.0-100.0)	0.46
FSH, IU/L	1.7 (0.8-2.5)	1.4 (0.9-2.7)	0.79
LH, IU/L	0.1 (0.1-0.1)	0.1 (0.1-0.1)	0.31
E2, pg/ml	5.0 (5.0-5.0)	5.0 (5.0-5.0)	0.79
PRL, ng/ml	11.9 (8.1-14.6)	11.0 (8.1-16.7)	0.42
T, ng/dl	2.5 (2.5-2.5)	2.5 (2.5-2.5)	0.38
TSH, uIU/ml	2.9 (2.1-3.4)	2.9 (2.3-4.4)	0.11
AMH, ng/ml^†^	4.9 (2.9-15.2)	4.5 (2.5-15.7)	0.82
DHEA-S, ug/dl	7.3 (3.3-14.3)	9.2 (3.9-21.3)	0.37
FT3, pmol/L	6.85 ± 0.87	6.8 ± 0.8	0.73
FT4, pmol/L	18.2 ± 1.9	18.1 ± 2.2	0.76
A-TG, IU/ml	10.8 (10.0-14.1)	10.7 (10.0-14.2)	0.80
A-TPO, IU/ml	8.4 (5.7-10.6)	8.6 (5.9-11.7)	0.63
BG0, mmol/L^†^	5.0 ± 0.4	4.9 ± 0.4	0.66
INS0, mIU/L^§^	4.3 (3.0-6.2)	4.4 (2.9-6.6)	0.47
HOMA-IR^§^	1.0 (0.7-1.3)	1.0 (0.6-1.6)	0.56
HOMA-β^§^	59.5 (40.0-81.2)	64.7 (50.9-96.5)	0.12
CHOL, mmol/L^†^	4.1 (3.6-4.8)	4.0 (3.7-4.3)	0.44
TG, mmol/L^†^	0.7 (0.6-0.9)	0.7 (0.6, 1.0)	0.33
LDL, mmol/L^†^	2.5 (2.1-3.0)	2.4 (2.1-2.7)	0.26
HDL, mmol/L^†^	1.3 ± 0.3	1.3 ± 0.2	0.93

Data were shown as mean ± SD, median (interquartile range).

Differences were assessed using Student t test or Mann-Whitney U tests for continuous variables and Chi-square tests for categorical variables.

a The linear regression was performed to exclude the potential confounding effects of maternal age and BMI [P-adjusted, 0.026; β (95%CI), -0.18 (-0.69, -0.05)].

^*^p<0.05 was considered statistically significant. ^†^represents a missing data. ^§^represents two missing data.

PCOS-off, offspring born to PCOS women; Con-off, offspring of the control group; BW, birth weight; GA, gestational age; BMI, body mass index; DBP, diastolic blood pressure; SBP, systolic blood pressure; FSH, follicle-stimulating hormone; LH, luteinizing hormone; E2, estrogen; PRL, prolactin; T, Testosterone; TSH, thyroid-stimulating hormone; AMH, anti-Mu ¨llerian hormone; DHEA-S, dehydroepiandrosterone sulfate; FT3, free triiodothyronine; FT4, free thyroxine; A-TG, antibodies against thyroglobulin; A-TPO, antibodies against thyroperoxidase; BG0, fasting glucose; INS0, fasting insulin; HOMA-IR, homeostasis model for insulin resistance; HOMA-β, homeostasis model for β-cell function; CHOL, total cholesterol; TG, triglycerides; LDL, low density lipoprotein cholesterol; HDL, high density lipoprotein cholesterol.

### Lipidomics

**Figure 1:**
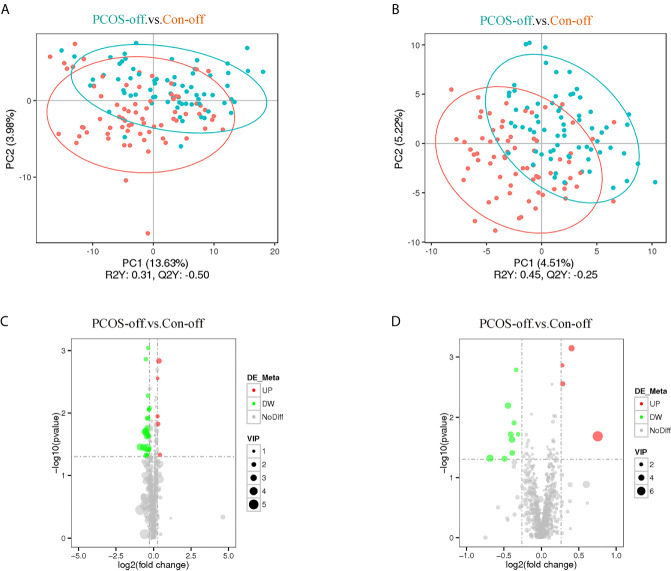
Plasma lipid profiles of the general offspring born to PCOS women (n_PCOS-off_ =70) or healthy control women (n_Con-off_ =71). **(A, B)** PLS-DA scores plot established the relationship model between the metabolite expression and the sample category, which indicated different metabolites profile between PCOS-off and Con-off in the positive **(A)** and negative **(B)** polarity modes. **(C, D)** Volcano plot of p values and fold change between PCOS-off and Con-off summarized the distribution of the differential lipids in the positive **(C)** and negative **(D)** polarity modes. P value was calculated using student t tests; VIP value represents variable importance in the projection value from PLS-DA model. Red, green, and gray dots represent metabolites with up-regulation, down-regulation, or no difference between study groups, respectively. PCOS-off, offspring born to PCOS women; Con-off, offspring of the control group.

Lipidomics analysis identified a total of 1779 metabolites. After deleting 8 metabolites expressed in less than 50% of samples, the remained metabolites for statistical analysis were 1771. As shown in **Figure 1**, plasma lipid profiles of the general offspring were revealed by the PLS-DA and volcano plot. The corresponding results for female and male offspring were shown in **Supplementary Figures 1** and **2**. PLS-DA scores plot showed different metabolite profiles between PCOS-off and control group. Volcanic map showed the overall distribution of differential metabolites. In total, we identified 8 categories of 44 metabolites significantly altered between the two groups in PCOS-off (**Table 3**), and 3 of which were previously reported having definite clinical relevance including glycerophospholipids (GP), glycerolipids (GL), and sphingomyelin (SM). Among them, 8 metabolites showed higher levels with 1 GP, 1 GL, and 1SM, and the other 36 metabolites showed lower levels with 6 GP and 19 GL.

**Table 3:** Metabolites altered between study groups in the general offspring.

Metabolites	VIP	FC	*P* value	Trend
Glycerolipids [GL]				
MG (0:0/20:4(8Z,11Z,14Z,17Z)/0:0)	1.51	1.33	0.05	↑
Monoelaidin	1.51	0.78	0.001	↓
DG (14:0/16:0/0:0)	2.26	0.75	0.02	↓
DG (14:0/18:2(9Z,12Z)/0:0)	1.66	0.77	0.01	↓
DG (14:1(9Z)/16:0/0:0)	2.95	0.68	0.02	↓
DG (15:0/18:1(11Z)/0:0)	4.59	0.62	0.05	↓
DG (16:0/16:1(9Z)/0:0)	2.27	0.71	0.05	↓
DG (16:1(9Z)/16:0/0:0)	1.42	0.78	0.02	↓
DG (16:1(9Z)/18:3(9Z,12Z,15Z)/0:0)	1.62	0.70	0.001	↓
TG (12:0/14:0/18:1(9Z)) [iso6]	3.18	0.54	0.04	↓
TG (12:0/16:0/18:1(9Z)) [iso6]	3.17	0.63	0.04	↓
TG (12:0/16:1(9Z)/18:2(9Z,12Z)) [iso6]	3.20	0.71	0.02	↓
TG (14:0/18:2(9Z,12Z)/16:1(9Z))	2.25	0.73	0.02	↓
TG (15:0/18:1(9Z)/16:1(9Z)) [iso6]	1.77	0.75	0.04	↓
TG (15:0/18:1(9Z)/18:1(9Z)) [iso3]	1.10	0.82	0.02	↓
TG (16:0/14:0/18:1(9Z)) [iso6]	2.14	0.73	0.05	↓
TG (16:0/14:0/18:2(9Z,12Z)) [iso6]	2.17	0.76	0.05	↓
TG (16:0/15:0/18:1(11Z))	2.24	0.76	0.01	↓
TG (16:1(9Z)/18:4(6Z,9Z,12Z,15Z)/18:1(11Z))	1.15	0.83	0.04	↓
TG (18:2(9Z,12Z)/14:0/18:3(9Z,12Z,15Z)) [iso6]	1.70	0.80	0.02	↓
Glycerophospholipids [GP]				
PA (P-16:0/18:1(9Z))	1.17	0.80	0.01	↓
PA (O-16:0/14:0)	1.05	0.77	0.02	↓
PC (o-18:0/22:0)	3.70	1.32	0.001	↑
** PC (13:0/0:0)**	3.97	0.76	0.02	↓
PI (18:3(9Z,12Z,15Z)/16:0)	3.93	0.73	0.01	↓
LysoPE (0:0/18:0)	2.15	0.81	0.02	↓
LysoPC (14:0)	3.45	0.75	0.02	↓
Fatty Acyls [FA]				
1-Tridecene	2.23	0.79	0.002	↓
2-Linoleoylglycerol	1.49	0.76	0.01	↓
cis-Uvariamicin IB	7.28	1.69	0.02	↑
Hexacosanoyl carnitine	2.19	1.29	0.001	↑
Methyl jasmonate	4.00	0.75	0.04	↓
Carboxylic acids and derivatives				
L-Glutamine	1.84	0.83	0.01	↓
Rhizonin A	1.48	0.80	0.01	↓
N-Nonanoylglycine	3.62	0.71	0.05	↓
Benzene and substituted derivatives				
3-Pentadecylphenol	1.43	1.20	0.01	↑
2-Hydroxybenzaldehyde	2.69	1.22	0.003	↑
Prenol Lipids [PR]				
Coenzyme Q10	1.12	1.20	0.003	↑
Sphingolipids [SP]				
** SM (d16:1/23:0)**	1.82	1.22	0.001	↑
Unclassified metabolites				
Endosulfan-sulfate	1.84	1.22	0.02	↑
bacteriohopane-31,32,33,34,35-pentol	2.97	0.70	0.02	↓
Progesterone, 16.alpha.-methyl-	1.05	0.82	0.04	↓
(24R)-25-fluoro-1alpha,24-dihydroxyvitamin D3	2.78	0.78	0.01	↓
12,13-DHOME	3.28	0.76	0.04	↓

The threshold value was VIP > 1.0, FC > 1.2 or FC < 0.833 and P value < 0.05.

The potential markers with clinical value were presented in bold.

VIP, variable importance in the projection value from PLS-DA model; FC, fold change; P value was calculated by student t test.

After stratification according to sex, in PCOS-g, 13 up-regulated metabolites (including 4 GP, 2 GL, and 7 other lipids) and 31 down-regulated ones (including 2 GP, 13 GL, and 16 other metabolites) were identified (**Table S3**). PCOS-b showed different lipids distribution presented as 9 up-regulated (1 SM and 8 others) and 35 down-regulated (16GP, 3GL, and 19 other species) (**Table S4**).

### Association Between Lipid Metabolites and Phenotypes

**Figure 2:**
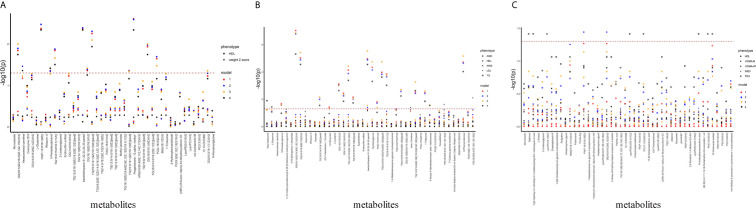
Manhattan plot showing significance of association between differential metabolites and clinical phenotypes. The x-axis represents metabolites differed between PCOS-off and con-off. The y-axis represents the -log_10_ p value. P value was obtained from the linear regression equation and then performed FDR correction. Dots with different shapes represent different phenotypes, and different colors represent different models. **(A)** Association between differential metabolites and clinical phenotypes in the general offspring (n_phenotype_=2, n_metabolites_=44). **(B)** Association between differential metabolites and clinical phenotypes in the female offspring (n_phenotype_=5, n_metabolites_=44). **(C)** Association between differential metabolites with clinical phenotypes in the male offspring (n_phenotype_=5, n_metabolites_=46). Glucoside^*^ refers to (1’x,2S)-2-(1,2-Dihydroxy-1-methylethyl)-2,3-dihydro-7H-furo[3,2-g][1]benzopyran-7-one 2’-glucoside.

In order to determine the associations of plasma lipids with clinical phenotypes, we screened the differential clinical phenotypes between the PCOS-off and the control offspring in this study, and combined them with those found in our previous studies and other studies as the outcome for linear regression analysis (12, 13). In summary, the outcome variables included weight Z-score, HDL (in PCOS-off); AMH, HDL, INS0, LDL, TG (in PCOS-g); HDL, HOMA-β, HOMA-IR, INS0, TSH (in PCOS-b), and independent variables included altered metabolites and other confounding factors. Manhattan blot showed original p value and p value after false discovery rate (FDR) adjustment in metabolite–phenotype multivariable linear regression analysis across different models (**Figure 2**).

After FDR correction and adjustment for all potential confounding factors (Model 4), in PCOS-off, GP and GL were correlated with HDL positively. In PCOS-g, GL were positively associated with TG, while GP was positively associated with LDL. In PCOS-b, in addition to the positive correlation between GP and TSH, no plasma lipid metabolites were significantly associated with HOMA-β, HOMA-IR, INS0, and HDL.

## Discussion

In this study, we investigated lipid profile changes in PCOS-off using a lipidomics approach based on UPLC-QTOF-MS. The results revealed significant alterations in GP, GL, SM, and some unclassified lipid metabolites. Furthermore, these changes were associated with differential phenotypes in all PCOS-off, including weight Z-score and HDL level, and showed sex-dependent correlation with TG, LDL, and TSH.

As the major component of mammalian biomembranes, GP participate in various biological pathways (19). In our clinical study, 16 metabolites were reduced in PCOS-b, including phosphatidylcholines (PC), lysophosphatidylcholine (lysoPC), phosphoethanolamine (PE), and lysophosphoethanolamine (lysoPE). PC can promote both cell proliferation and programmed cell death and is known to improve insulin sensitivity (20). LysoPC is a compound produced by hydrolysis of PC, and an accumulating number of studies show that it plays a vital role in glucose homeostasis. Plasma lysoPC is decreased in T2D and obesity (21). LysoPC has also been shown to stimulate adipocyte glucose uptake and improve blood glucose levels in murine models of diabetes (22); furthermore, exogenous lysoPC can inhibit free fatty acid-induced C-Jun N-terminal kinase activation and insulin resistance (23). An additional study found that lysoPC could induce PKC-α activation and inhibit Akt activation in vascular smooth muscle cells (VSMCs) by blocking IRS-1 function suggesting a role for lysoPC in vascular insulin resistance (24). The reduction of PC and lysoPC in PCOS-b may indicate an increased risk of insulin resistance and diabetes mellitus, which was consistent with our previous study that found aberrant insulin metabolism in PCOS-b (12). However, in the present study we found no correlation between changes in GP and glucose metabolic parameters, instead, an association of GP with TSH was indicated. That may be owing to the bilateral changes of the components. The association of specific lipids, such as PC and lypoPC, with glucose metabolism in PCOS-off is worthy of future study.

SM was another identified metabolite that was significantly changed in PCOS-off in the present study. As one of the major phospholipids in the lipid microdomains it was found to be positively correlated with insulin resistance. SM synthase 2 knock-out mice have been shown to escape from high fat diet-induced obesity and insulin resistance, while silencing of SM synthase 2 is able to reduce lipid droplets in the liver (25). Lipid rafts, which are highly enriched in SM, are thought to be essential regulators of insulin signaling (26); although the mechanism still needs to be explained through future studies. Furthermore, plasma SM mainly exists in atherogenic lipoproteins, and is correlated with the metabolism of apoB-containing or TG-rich lipoproteins (27). SM carried into the arterial wall on atherogenic lipoproteins can promote lipoprotein aggregation (28). SM has also been shown to be associated with subclinical atherosclerosis and coronary artery disease (27, 29). Therefore, our finding that increased SM levels in PCOS-off indicate an increased risk of aberrant cardiometabolic health show that SM should be carefully monitored.

The results of our study showed a significant decrease of GL, such as diglyceride (DG) and TG, in PCOS-g. TG and DG are reported to be associated with impaired glucose tolerance, insulin resistance, and T2D (30, 31). In plasma, elevated proportions of TG and DG are positively correlated with liver fat, visceral fat, systolic blood pressure, and insulin resistance (30). Our finding of the decreased levels of GL in PCOS-g may indicate the lower risk of insulin resistance, diabetes, and obesity, which was consistent with some recent studies. Li et al. (12) compared PCOS-g with normal girls and found no difference in glucose or lipid profiles. A further individual participant data meta-analysis indicated a healthy performance of lipids in PCOS-g, including decreased LDL and CHOL, as well as increased HDL levels (13). Protection of E2 may be an explanation. The correlation between GL components and serum TG levels was also confirmed in the present study which indicated the possible protective effect of E2 on lipid profiles. However, the meta-analysis showed increased after-loaded insulin levels in PCOS-g, which was contradictory with changes in metabolites (13). Similarly, PCOS women also show reduced GL levels (32), which may be explained by differential lipid structure. Lipids with lower carbon number and double bond content have been found to be associated with a higher risk of diabetes and vice versa (33). Furthermore, PCOS subtypes may also have varied lipid profiles. Finally, obese PCOS women show increased lipolysis, while nonobese PCOS women present with decreased lipolysis and enhanced glucose utilization in peripheral tissues (34). Therefore, the effect of changes in GL level on PCOS-g remain to be elucidated.

Strengths of the current study include its comprehensive view of lipid profiles in PCOS-off, generated using a lipidomics approach, and its identification of sex specific changes of the lipid metabolites. A series of changes were found in the metabolism and endocrinology of PCOS-off, such as birth weight, HDL, AMH, and TSH, some of which differed according to sex (12, 13). The results built a link between biomolecular changes and clinical phenotypes, which suggested potential sensitive markers for the early detection of aberrant cardiometabolic events in this well-accepted susceptible population, along with new indications in pathogenesis studies of relative disorders. However, our study also had several limitations. Firstly, the relatively small sample size after stratification according to sex limited the statistical power. Secondly, carboxylic acids and derivatives, as well as PR and some unclassified metabolites also showed significant changes in PCOS-off, and some of them presented with sex differences. However, the category and function of these metabolites were not detected and their potential impact was not clear. The mechanism of these metabolites involved in PCOS related phenotypic changes needs to be further explored. Although hundreds of differential lipids were found in this lipidomics analysis, other species of metabolites are not included and await further broader studies. Moreover, although we adjusted some confounding factors, other potential confounding maternal factors, such as smoking, drinking, and breastfeeding, were not included in the present study owing to the lack of data. Furthermore, the number of cases of some confounding factors, such as GDM, were relatively small, which may limit the statistical power in multiple regression analysis.

In conclusion, we observed that PCOS-off already presented specific lipid profile alterations during childhood, before significant differences were detected by regular clinical metabolic screening tests. The abnormal level of GP and SM indicated the risk of glucose metabolism and cardiovascular diseases in PCOS-off. This susceptible population therefore needs more specific and detailed monitoring and management. Lipid metabolites, such as PC, lysoPC, and SM identified using rapid detection kits, are suggested as potential markers. Their pathogenic roles need to be elucidated in future functional studies.

## Data Availability Statement

The raw data supporting the conclusions of this article will be made available by the authors, without undue reservation.

## Ethics Statement

The studies involving human participants were reviewed and approved by The institutional ethics committee of Shandong University. Written informed consent to participate in this study was provided by the participants’ legal guardian/next of kin.

## Author Contributions

HZ and LC designed the study. YL selected the study population and collected plasma samples. ZZ and YL drafted the manuscript. DZ, JLi, KH and JM collected clinical data used in the study. JLv performed statistical analysis. HZ, LC, ZZ and YL participated in the revision and final approval of the manuscript. All authors contributed to the article and approved the submitted version.

## Funding

This work was supported by The National Key Research and Development Program of China 2016YFC1000200 and 2016YFC10002004, 2018YFC1004301, Basic Science Center Program of NSFC 31988101, Shandong Provincial Key Research and Development Program 2018YFJH0504, and Shandong Province Medical and Health Technology Development Project 2016WS0368.The sponsors had no role in this study.

## Conflict of Interest

The authors declare that the research was conducted in the absence of any commercial or financial relationships that could be construed as a potential conflict of interest.

## Publisher’s Note

All claims expressed in this article are solely those of the authors and do not necessarily represent those of their affiliated organizations, or those of the publisher, the editors and the reviewers. Any product that may be evaluated in this article, or claim that may be made by its manufacturer, is not guaranteed or endorsed by the publisher.

## Abbreviations

PCOS, polycystic ovary syndrome; PCOS-off, offspring born to PCOS women**;** PCOS-g, girls born to PCOS women**;** PCOS-b, boys born to PCOS women**;** TG, triglyceride**;** LDL, low density lipoprotein cholesterol**;** HDL, high density lipoprotein cholesterol**;** PC, phosphatidylcholines**;** lysoPC, lysophosphatidylcholine**;** SM, sphingomyelin; T2D, type 2 diabetes; PIH, pregnancy-induced hypertension**;** GDM, gestational diabetes mellitus; CHOL, cholesterol; DBP, diastolic blood pressure; SBP, systolic blood pressure**;** FSH, follicle-stimulating hormone; LH, luteinizing hormone**;** E2, estrogen; PRL, prolactin**;** T, testosterone; TSH, thyroid-stimulating hormone**;** AMH, anti-Mu ¨llerian hormone**;** DHEA-S, dehydroepiandrosterone sulfate**;** FT3, free triiodothyronine; FT4, free thyroxine**;** A-TG, antibodies against thyroglobulin; A-TPO, antibodies against thyroperoxidase**;** BG0, fasting glucose; INS0, fasting insulin**;** HOMA-IR, homeostasis model for insulin resistance**;** HOMA-β, homeostasis model for β-cell function**;** PCA, principal component analysis**;** PLS-DA, partial least-squares discrimination analysis; VIP, variable importance in the projection; FC, fold change**;** FDR, false discovery rate**;** GL, glycerolipids**;** GP, glycerophospholipids**;** FA, fatty acyls**;** PR, prenol**;** SP, sphingolipids**;** PE, phosphoethanolamine**;** lysoPE, lyso phosphoethanolamine; VSMCs, vascular smooth muscle cells.
