# Supplementary material for: Differential Lipidomic Characteristics of Children Born to Women with Polycystic Ovary Syndrome
Source: Front Endocrinol (Lausanne). 2021 Aug 9;12:698734. doi: 10.3389/fendo.2021.698734 (PMC8380809; doi:10.3389/fendo.2021.698734)
Supplement: Supplementary file 1 [file DataSheet_1.docx]

Supplementary Material


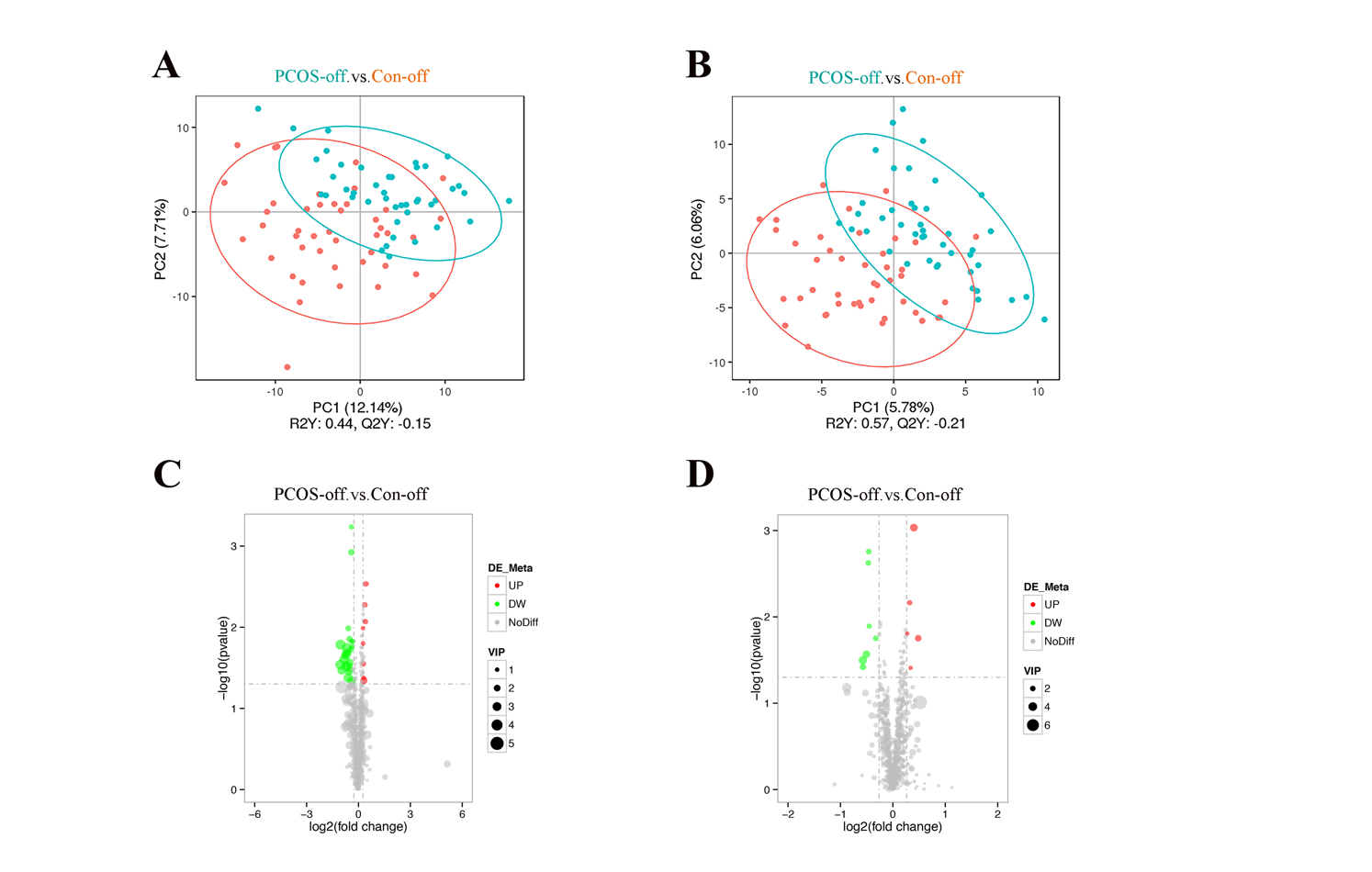


**Supplementary Figure 1.** Plasma lipid profiles of the female offspring born to PCOS women (n_PCOS-off_ =43) or healthy control women (n_Con-off_ =44). For explanation of the plot, see Figure. 1 legend


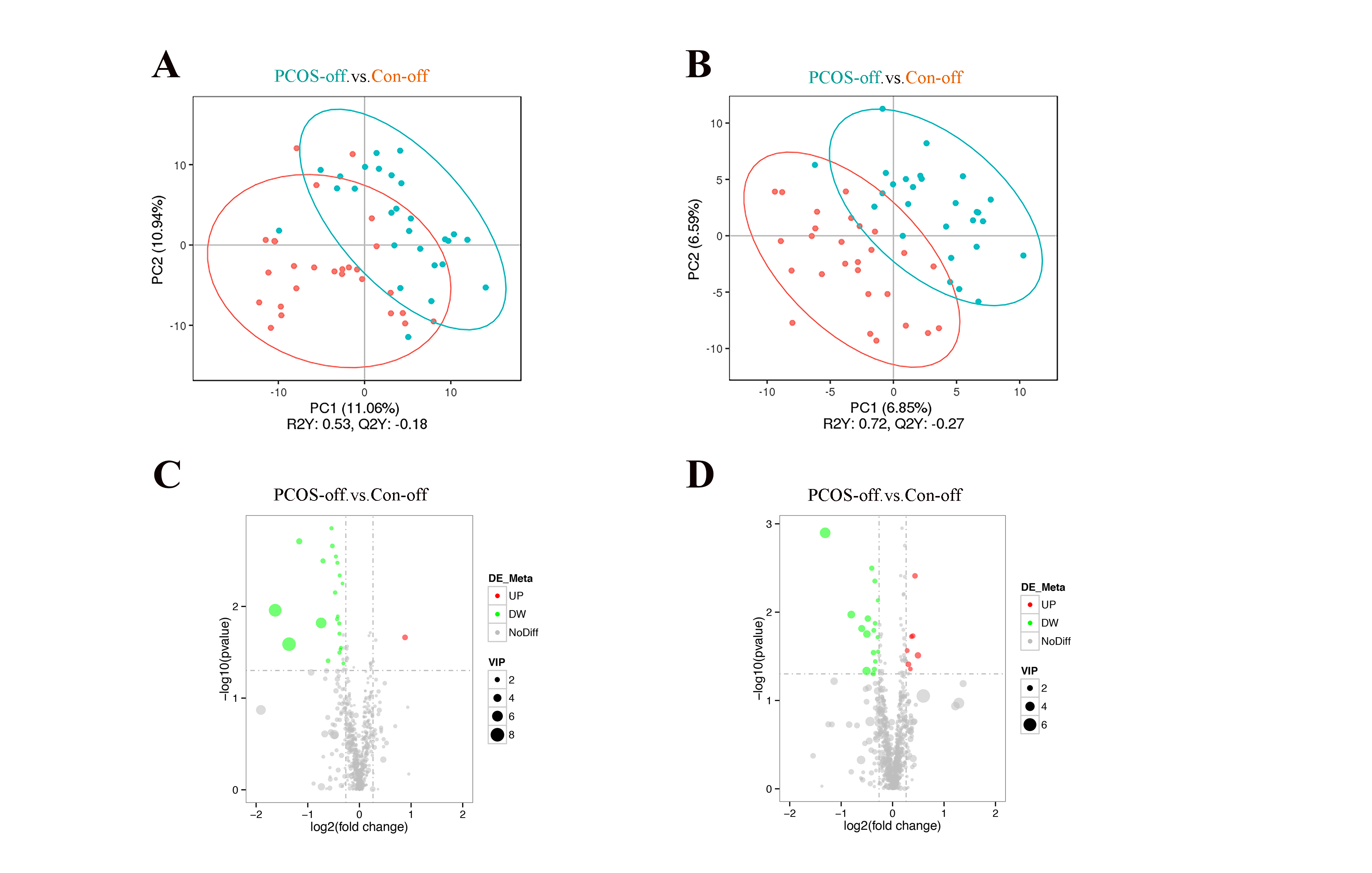


**Supplementary Figure 2.** Plasma lipid profiles of the male offspring born to PCOS women (n_PCOS-off_ =27) or healthy control women (n_Con-off_ =27). For explanation of the plot, see Figure. 1 legend

**Table S1. Clinical characteristics of the female offspring born to women with or without PCOS**

| **Characteristics** | **PCOS-off (n=43)** | **Con-off (n=44)** | ***P* value** |
| --- | --- | --- | --- |
| Age, yrs | 3.6 (3.2-5.0) | 3.6 (3.1-5.0) | 0.52 |
| BW, g | 3405.2±543.4 | 3409.8±462.9 | 0.97 |
| GA, week | 40.0 (39.0-40.0) | 39.0 (38.0-40.0) | 0.39 |
| Height Z-score | 0.3±0.9 | 0.4±0.8 | 0.40 |
| Weight Z-score | 0.2±0.9 | 0.6±1.0 | 0.09 |
| BMI Z-score | 0.2 (-0.5-0.5) | 0.4 (-0.6-0.9) | 0.18 |
| DBP, mmHg | 58.0 (54.0-62.0) | 57.0 (54.0-60.0) | 0.28 |
| SBP, mmHg | 90.0 (88.0-96.0) | 90.0 (86.5-99.5) | 0.84 |
| FSH, IU/L | 2.2 (1.7-2.9) | 1.9 (1.4-3.3) | 0.82 |
| LH, IU/L | 0.1 (0.1-0.1) | 0.1 (0.1-0.1) | 0.16 |
| E2, pg/ml | 5.0 (5.0-5.0) | 5.0 (5.0-5.0) | 0.88 |
| PRL, ng/ml | 12.6 (8.1-14.8) | 10.9 (8.1-16.4) | 0.67 |
| T, ng/dl | 2.5 (2.5-2.5) | 2.5 (2.5-2.5) | 0.29 |
| TSH, uIU/ml | 3.0 (2.3-3.6) | 2.7 (2.2-4.0) | 0.95 |
| AMH, ng/ml | 3.3 (1.8-4.7) | 2.8 (1.3-4.3) | 0.33 |
| DHEA-S, ug/dl | 8.3 (3.9-16.2) | 8.8 (5.0-19.9) | 0.69 |
| FT3, pmol/L | 6.8±0.9 | 6.9±0.8 | 0.77 |
| FT4, pmol/L | 18.0±2.0 | 18.4±2.1 | 0.46 |
| A-TG, IU/ml | 11.1 (10.0-14.0) | 11.3 (10.0-15.2) | 0.71 |
| A-TPO, IU/ml | 9.0 (6.7-11.3) | 8.2 (6.2-12.7) | 0.75 |
| BG0, mmol/L | 5.0±0.4 | 4.9±0.4 | 0.38 |
| INS0, mIU/L | 4.3 (3.0-6.5) | 4.1 (2.8-6.2) | 0.90 |
| HOMA-IR | 1.0 (0.7-1.4) | 0.9 (0.5-1.5) | 0.99 |
| HOMA-β | 61.6 (42.0-94.7) | 61.8 (48.1-98.1) | 0.37 |
| CHOL, mmol/L | 4.3 (3.8-4.8) | 4.1 (3.6-4.3) | 0.10 |
| TG, mmol/L | 0.7 (0.6-0.9) | 0.7 (0.6-0.9) | 0.79 |
| LDL, mmol/L | 2.6 (2.2-3.2) | 2.4 (2.1-2.7) | 0.06 |
| HDL, mmol/L | 1.3±0.3 | 1.3±0.2 | 0.75 |
| Data were shown as mean ± SD, median (interquartile range).  Differences were assessed using Student t test or Mann-Whitney U tests for continuous variables and Chi-square tests for categorical variables.  p<0.05 was considered statistically significant.  The meaning of abbreviations can be found in Table 2. | | | |

**Table S2 Clinical characteristics of the male offspring born to women with or without PCOS**

| **Characteristics** | **PCOS-off (n=27)** | **Con-off (n=27)** | ***P* value** |
| --- | --- | --- | --- |
| Age, yrs | 3.4 (3.2-5.3) | 3.6 (3.3-5.1) | 0.36 |
| BW, g | 3402.0±622.6 | 3445.2±425.9 | 0.77 |
| GA, week | 39.0 (38.0-40.0) | 39.0 (38.0-40.0) | 0.39 |
| Height Z-score | 0.4±0.9 | 0.6±1.0 | 0.47 |
| Weight Z-score | 0.5±1.1 | 0.9±1.3 | 0.18 |
| BMI Z-score | 0.4 (-0.5-0.7) | 0.5 (-0.5-1.9) | 0.26 |
| DBP, mmHg^†^ | 56.0 (53.5-60.0) | 58.0 (52.0-64.0) | 0.87 |
| SBP, mmHg^†^ | 91.0 (88.0-98.0) | 94.0 (88.0-102.0) | 0.33 |
| FSH, IU/L | 0.8 (0.5-1.1) | 0.9 (0.7-1.2) | 0.47 |
| LH, IU/L | 0.1 (0.1-0.1) | 0.1 (0.1-0.1) | 0.73 |
| E2, pg/ml | 5.0 (5.0-5.0) | 5.0 (5.0-5.0) | 0.33 |
| PRL, ng/ml | 9.6 (8.1-13.4) | 11.0 (8.1-17.8) | 0.42 |
| T, ng/dl | 2.5 (2.5-2.5) | 2.5 (2.5-2.5) | 0.98 |
| TSH, uIU/ml^a^ | 2.3 (1.9-3.2) | 3.5 (2.3-5.4) | **0.03^*^** |
| AMH, ng/ml^†^ | 15.5 (14.9-16.5) | 15.7 (15.3-16.6) | 0.25 |
| DHEA-S, ug/dl | 5.5 (2.3-14.1) | 12.1 (2.3-26.1) | 0.39 |
| FT3, pmol/L | 6.9±0.9 | 6.7±0.8 | 0.36 |
| FT4, pmol/L | 18.6±1.8 | 17.8±2.4 | 0.18 |
| A-TG, IU/ml | 10.1 (10.0-14.5) | 10.0 (10.0-12.2) | 0.34 |
| A-TPO, IU/ml | 6.9 (5.1-9.3) | 8.8 (5.0-10.6) | 0.26 |
| BG0, mmol/L^†^ | 4.9±0.4 | 5.0±0.4 | 0.72 |
| INS0, mIU/L^§^ | 4.4 (3.0-5.5) | 4.7 (3.4-7.3) | 0.33 |
| HOMA-IR^§^ | 0.9 (0.7-1.2) | 1.0 (0.7-1.7) | 0.37 |
| HOMA-β^§^ | 54.9 (37.6-76.9) | 68.8 (52.1-96.5) | 0.16 |
| CHOL, mmol/L^†^ | 3.8 (3.2-4.7) | 4.0 (3.7-4.3) | 0.37 |
| TG, mmol/L^†^ | 0.7 (0.6-0.8) | 0.7 (0.7-1.0) | 0.20 |
| LDL, mmol/L^†^ | 2.3 (2.0-2.8) | 2.4 (2.1-2.7) | 0.49 |
| HDL, mmol/L^†^ | 1.4±0.2 | 1.3±0.2 | 0.58 |
| Data were shown as mean ± SD, median (interquartile range).  Differences were assessed using Student t test or Mann-Whitney U tests for continuous variables and Chi-square tests for categorical variables.  ^a^ The linear regression was performed to exclude the potential confounding effects of maternal age and BMI (P-adjusted, 0.03; β (95%CI), -0.30 (-1.13, -0.06)).  ^*^ p<0.05 was considered statistically significant. ^†^ represents a missing data. ^§^ represents two missing data.  The meaning of abbreviations can be found in Table 2. | | | |

**Table S3. Metabolites altered between study groups in the female offspring**

| **Metabolites** | **VIP** | **FC** | ***P* value** | **Trend** |
| --- | --- | --- | --- | --- |
| Glycerolipids [GL] |  |  |  |  |
| 1-(14-methyl-pentadecanyl)-2-(8-[3]-ladderane-octanyl)-sn-glycerol | 1.42 | 1.33 | 0.01 | ↑ |
| MG (0:0/22:1(13Z)/0:0) | 3.36 | 0.71 | 0.03 | ↓ |
| DG (14:0/16:0/0:0) | 2.29 | 0.65 | 0.02 | ↓ |
| DG (14:0/18:2(9Z,12Z)/0:0) | 1.94 | 0.70 | 0.01 | ↓ |
| DG (14:1(9Z)/16:0/0:0) | 3.41 | 0.62 | 0.02 | ↓ |
| DG (16:1(9Z)/0:0/16:1(9Z)) (d5) | 1.13 | 1.23 | 0.04 | ↑ |
| DG (16:1(9Z)/18:3(9Z,12Z,15Z)/0:0) | 1.70 | 0.67 | 0.01 | ↓ |
| TG (12:0/14:0/18:1(9Z)) [iso6] | 3.62 | 0.49 | 0.02 | ↓ |
| TG (12:0/16:0/18:1(9Z)) [iso6] | 3.52 | 0.57 | 0.03 | ↓ |
| TG (12:0/16:0/18:2(9Z,12Z)) [iso6] | 3.23 | 0.66 | 0.04 | ↓ |
| TG (12:0/16:1(9Z)/18:2(9Z,12Z)) [iso6] | 3.66 | 0.62 | 0.03 | ↓ |
| TG (12:0/18:2(9Z,12Z)/18:3(6Z,9Z,12Z)) [iso6] | 3.13 | 0.76 | 0.05 | ↓ |
| TG (14:0/15:0/15:0) | 2.85 | 0.69 | 0.04 | ↓ |
| TG (14:0/18:2(9Z,12Z)/16:1(9Z)) | 2.55 | 0.58 | 0.02 | ↓ |
| TG (16:0/15:0/18:1(11Z)) | 2.09 | 0.70 | 0.03 | ↓ |
| Glycerophospholipids [GP] |  |  |  |  |
| PA (o-16:0/14:0) | 1.25 | 0.73 | 0.03 | ↓ |
| PC (o-18:0/22:0) | 3.46 | 1.32 | 0.001 | ↑ |
| PC (22:1(13Z)/18:0) | 1.94 | 1.25 | 0.01 | ↑ |
| PI (18:3(9Z,12Z,15Z)/16:0) | 2.67 | 0.67 | 0.04 | ↓ |
| PG (16:0/22:4(7Z,10Z,13Z,16Z)) | 1.39 | 1.26 | 0.04 | ↑ |
| PG (18:0/22:4(7Z,10Z,13Z,16Z)) | 1.37 | 1.21 | 0.02 | ↑ |
| Fatty Acyls [FA] |  |  |  |  |
| 1-Tridecene | 2.18 | 0.73 | 0.002 | ↓ |
| Hexacosanoyl carnitine | 1.55 | 1.35 | 0.003 | ↑ |
| Carboxylic acids and derivatives |  |  |  |  |
| Myristoylglycine | 2.18 | 0.60 | 0.03 | ↓ |
| Valyl-Isoleucine | 1.38 | 0.75 | 0.001 | ↓ |
| L-Glutamine | 1.90 | 0.75 | 0.001 | ↓ |
| Rhizonin A | 1.42 | 0.79 | 0.02 | ↓ |
| L-Lysopine | 2.01 | 0.73 | 0.02 | ↓ |
| Benzene and substituted derivatives |  |  |  |  |
| 3-Pentadecylphenol | 1.53 | 1.29 | 0.01 | ↑ |
| Peonidin | 1.19 | 1.20 | 0.02 | ↑ |
| Sphingolipids [SP] |  |  |  |  |
| C16 Sphinganine-1-phosphate | 1.06 | 1.20 | 0.01 | ↑ |
| Benzopyrans |  |  |  |  |
| 7'-Carboxy-gamma-tocotrienol | 2.75 | 1.40 | 0.02 | ↑ |
| Unclassified metabolites |  |  |  |  |
| Alprenolol | 1.36 | 0.76 | 0.02 | ↓ |
| Guanethidine | 2.52 | 0.70 | 0.03 | ↓ |
| (Z)-7-Hexadecen-1,16-olide | 1.33 | 0.77 | 0.02 | ↓ |
| 1,2-Dimyristin | 3.61 | 0.63 | 0.02 | ↓ |
| Endosulfan-sulfate | 1.41 | 1.22 | 0.03 | ↑ |
| bacteriohopane-31,32,33,34,35-pentol | 3.49 | 0.48 | 0.03 | ↓ |
| 1,2-Ditetradecanoyl-sn-glycero-3-phosphocholine | 2.95 | 0.51 | 0.03 | ↓ |
| .beta.-Carotene | 2.33 | 1.24 | 0.05 | ↑ |
| 5-Deoxy-5-(dimethylarsinoyl)-.beta.-ribofuranose | 2.11 | 0.72 | 0.002 | ↓ |
| Fensulfothion | 1.88 | 0.73 | 0.01 | ↓ |
| 24-ethyl-5alpha-cholest-25-en-3alpha,12alpha,16alpha-triol | 2.15 | 0.80 | 0.02 | ↓ |
| 6-Phenylundecane | 3.86 | 0.67 | 0.03 | ↓ |
| The threshold value was VIP > 1.0, FC > 1.2 or FC < 0.833 and P value < 0.05.  VIP, variable importance in the projection value from PLS-DA model; FC, fold change; P value was calculated by student t test. | | | | |

**Table S4. Metabolites altered between study groups in the male offspring**

| **Metabolites** | **VIP** | **FC** | ***P* value** | **Trend** |
| --- | --- | --- | --- | --- |
| Glycerolipids [GL] |  |  |  |  |
| Monoelaidin | 1.41 | 0.69 | 0.001 | ↓ |
| MG (0:0/18:1(11Z)/0:0) | 1.12 | 0.75 | 0.01 | ↓ |
| DG (18:0/18:2(9Z,12Z)/0:0) | 1.11 | 0.78 | 0.03 | ↓ |
| Glycerophospholipids [GP] |  |  |  |  |
| 1-(9Z-Octadecenoyl)-sn-glycero-3-phosphocholine | 1.03 | 0.80 | 0.01 | ↓ |
| 1-Heptadecanoyl-sn-glycero-3-phosphocholine | 1.27 | 0.76 | 0.02 | ↓ |
| PA (P-16:0/18:1(9Z)) | 1.41 | 0.76 | 0.02 | ↓ |
| **PC (13:0/0:0)** | 3.50 | 0.70 | 0.05 | ↓ |
| **PC (16:1(9Z)/22:6(4Z,7Z,10Z,13Z,16Z,19Z))** | 1.17 | 0.81 | 0.04 | ↓ |
| **PC (17:1(10Z)/0:0)** | 1.16 | 0.82 | 0.01 | ↓ |
| PE (19:0/0:0) | 1.66 | 0.78 | 0.04 | ↓ |
| PE (21:0/0:0) | 1.53 | 0.78 | 0.02 | ↓ |
| PE (P-16:0e/0:0) | 1.57 | 0.79 | 0.004 | ↓ |
| PE (O-20:0/0:0) | 1.34 | 0.79 | 0.04 | ↓ |
| PS (P-20:0/0:0) | 1.20 | 0.82 | 0.02 | ↓ |
| **LysoPC (16:1(9Z))** | 1.34 | 0.74 | 0.003 | ↓ |
| **LysoPC (18:1(9Z))** | 1.39 | 0.79 | 0.01 | ↓ |
| **LysoPC (22:4(7Z,10Z,13Z,16Z))** | 1.14 | 0.78 | 0.03 | ↓ |
| LysoPE (0:0/20:1(11Z)) | 1.75 | 0.76 | 0.003 | ↓ |
| LysoPE (0:0/24:1(15Z)) | 1.19 | 0.82 | 0.03 | ↓ |
| Fatty Acyls [FA] |  |  |  |  |
| 2-Linoleoylglycerol | 2.04 | 0.61 | 0.003 | ↓ |
| 10-Undecenyl acetate | 2.01 | 1.24 | 0.04 | ↑ |
| Palmitic acid | 1.77 | 0.66 | 0.04 | ↓ |
| Carboxylic acids and derivatives |  |  |  |  |
| L-Proline | 1.29 | 0.73 | 0.003 | ↓ |
| Benzene and substituted derivatives |  |  |  |  |
| 2-Hydroxybenzaldehyde | 1.90 | 1.35 | 0.004 | ↑ |
| N-Undecylbenzenesulfonic acid | 1.80 | 0.77 | 0.03 | ↓ |
| Prenol Lipids [PR] |  |  |  |  |
| Pubescenol | 1.40 | 0.74 | 0.01 | ↓ |
| 4-(4-Methyl-3-pentenyl)-3-cyclohexene-1-carboxaldehyde | 1.71 | 0.77 | 0.05 | ↓ |
| Sphingolipids [SP] |  |  |  |  |
| **SM (d16:1/23:0)** | 1.35 | 1.27 | 0.04 | ↑ |
| Sterol Lipids [ST] |  |  |  |  |
| 2-deoxy-20-hydroxy-5alpha-ecdysone 3-acetate | 1.40 | 0.76 | 0.03 | ↓ |
| Organothiophosphorus compounds |  |  |  |  |
| Mecarbam | 1.77 | 1.31 | 0.02 | ↑ |
| Cytochalasans |  |  |  |  |
| Chaetoglobosin N | 1.25 | 0.77 | 0.01 | ↓ |
| Unclassified metabolites |  |  |  |  |
| Rotigotine | 1.57 | 0.72 | 0.01 | ↓ |
| Hexapropylene glycol | 7.54 | 0.32 | 0.01 | ↓ |
| Trp-Ile-Arg | 6.09 | 0.60 | 0.02 | ↓ |
| 11alpha-(4-dimethylaminophenyl)-1alpha,25-dihydroxyvitamin D3 | 2.48 | 1.84 | 0.02 | ↑ |
| Heptapropylene glycol | 8.13 | 0.39 | 0.03 | ↓ |
| 12,13-DHOME | 4.82 | 0.40 | 0.001 | ↓ |
| 17,20-hexacosadienoic acid | 3.16 | 0.57 | 0.01 | ↓ |
| 6,8-Tricosanedione | 2.45 | 0.72 | 0.01 | ↓ |
| (24R)-25-fluoro-1alpha,24-dihydroxyvitamin D3 | 2.78 | 0.66 | 0.02 | ↓ |
| Aliskiren | 3.04 | 0.71 | 0.02 | ↓ |
| (1'x,2S)-2-(1,2-Dihydroxy-1-methylethyl)-2,3-dihydro-7H-furo[3,2-g][1]benzopyran-7-one 2'-glucoside | 1.48 | 1.29 | 0.02 | ↑ |
| 2,5-Dimethyl-1,4-dithiane-2,5-diol | 1.50 | 1.22 | 0.03 | ↑ |
| (2Z,6E)-3,7,11,15,19-Pentamethyl-2,6-eicosadien-1-ol | 2.39 | 1.41 | 0.03 | ↑ |
| Nabilone | 2.78 | 0.45 | 0.002 | ↓ |
| 7-[(6-Hydroxy-3,7-dimethyl-2,7-octadienyl)oxy]-2H-1-benzopyran-2-one | 1.78 | 0.70 | 0.002 | ↓ |
| The threshold value was VIP > 1.0, FC > 1.2 or FC < 0.833 and P value < 0.05.  The potential markers with clinical value were presented in bold.  VIP, variable importance in the projection value from PLS-DA model; FC, fold change; P value was calculated by student t test. | | | | |
